# Alcohol’s Effects on the Brain: Neuroimaging Results in Humans and Animal Models

**DOI:** 10.35946/arcr.v38.2.04

**Published:** 2017

**Authors:** Natalie M. Zahr, Adolf Pfefferbaum

**Affiliations:** Natalie M. Zahr, Ph.D., is a Research Scientist in the Department of Psychiatry and Behavioral Sciences, Stanford University School of Medicine, Stanford, California; and Program Director of Translational Imaging, Neuroscience Program, SRI International, Menlo Park, California. Adolf Pfefferbaum, M.D., is Professor of Psychiatry and Behavioral Sciences at Stanford University School of Medicine, Stanford, California; and Distinguished Scientist and Center Director of the Neuroscience Program, SRI International, Menlo Park, California

**Keywords:** Magnetic resonance imaging, diffusion tensor imaging, MR spectroscopy, alcohol use disorder, thiamine, liver, cerebellum

## Abstract

Brain imaging technology has allowed researchers to conduct rigorous studies of the dynamic course of alcoholism through periods of drinking, sobriety, and relapse and to gain insights into the effects of chronic alcoholism on the human brain. Magnetic resonance imaging (MRI) studies have distinguished alcohol-related brain effects that are permanent from those that are reversible with abstinence. In support of postmortem neuropathological studies showing degeneration of white matter, MRI studies have shown a specific vulnerability of white matter to chronic alcohol exposure. Such studies have demonstrated white-matter volume deficits as well as damage to selective gray-matter structures. Diffusion tensor imaging (DTI), by permitting microstructural characterization of white matter, has extended MRI findings in alcoholics. MR spectroscopy (MRS) allows quantification of several metabolites that shed light on brain biochemical alterations caused by alcoholism. This article focuses on MRI, DTI, and MRS findings in neurological disorders that commonly co-occur with alcoholism, including Wernicke’s encephalopathy, Korsakoff’s syndrome, and hepatic encephalopathy. Also reviewed are neuroimaging findings in animal models of alcoholism and related neurological disorders. This report also suggests that the dynamic course of alcoholism presents a unique opportunity to examine brain structural and functional repair and recovery.

Apart from direct effects on the brain, excessive alcohol consumption is associated with increased risk for trauma (i.e., traumatic brain injury) (Alterman and Tarter 1985; Chen et al. 2012), seizures (Eyer et al. 2011; Martindale et al. 2011), and stroke (de los Rios et al. 2012; Suzuki and Izumi 2013), each of which can have effects on brain structure independent of alcohol or each other. Furthermore, alcohol can alter the brain by affecting peripheral organs, including the digestive tract (e.g., Bienia et al. 2002; Duell et al. 2012), liver (e.g., Cederbaum 2012), heart (e.g., Roerecke et al. 2011), pancreas (e.g., Andersen et al. 2008), kidneys (e.g., Schaeffner and Ritz 2012), and lungs (e.g., Yeligar et al. 2012). Mechanisms of these indirect effects of alcohol on the brain are likely mediated via soluble factors, such as ceramides (e.g., de la Monte et al. 2012).

To evaluate alcohol’s central nervous system effects, researchers distinguish “uncomplicated alcoholism” (i.e., alcohol use disorder [AUD]) from the various clinically diagnosable consequences of chronic alcohol consumption, including Wernicke’s encephalopathy (WE), Korsakoff’s syndrome (KS), hepatic encephalopathy (HE), central pontine myelinolysis (CPM), alcoholic cerebellar degeneration (ACD), alcohol-related dementia (ARD), and Marchiafava-Bignami disease (MBD).^1^ The use of brain-imaging technology to evaluate clinically defined syndromes associated with chronic alcoholism, each with relatively unique radiological signatures (see table 1 and figure 1), provides guideposts for studying brain alterations associated with uncomplicated alcoholism.

Approximately 7 percent of adults age 18 and older have an AUD (Substance Abuse and Mental Health Services Administration 2013). Prevalence estimates of alcoholism-related syndromes are difficult to ascertain. Incidence estimates often are based on postmortem findings. Postmortem evaluation indicates a prevalence of 2 percent of WE in the general population; however, as many as 12 to 18 percent of alcoholics can have postmortem evidence of WE (Harper et al. 1988; Riethdorf et al. 1991; Thomson et al. 2002). Based on observations that 80 to 85 percent of patients with WE can develop KS, the estimated prevalence of KS is 11 to 12 percent of the alcoholic population (Day et al. 2013; Victor et al. 1971). Wernicke-Korsakoff syndrome (WKS) is used to refer to the presence of both WE and KS because of the close relationship between the two disorders.

Estimates of HE are derived from estimates of alcoholic cirrhosis, which can range from 8 percent to 20 percent (Bellentani et al. 1997; Mann et al. 2003; Sorensen et al. 1984). Mild HE occurs in up to 80 percent of cirrhotic patients, and overt HE occurs in up to 45 percent of cirrhotic patients (Bajaj 2008; Poordad 2007). One study estimated the incidence of CPM at 0.5 percent among the general population (Newell and Kleinschmidt-DeMasters 1996). However, prevalence is much higher (30 percent) among patients with liver transplants (Singh et al. 1994). For ACD prevalence, reports based on postmortem evaluation range from as low as 0.4 percent to as high as 42 percent of alcoholics (Riethdorf et al. 1991; Scholz et al. 1986; Stork 1967; Torvik and Torp 1986). Rates of ARD can depend on the setting, with facilities specializing in early identification and treatment of memory disorders reporting rates of 3 percent (McMurtray et al. 2006) and nursing homes reporting rates as high as 24 percent (Carlen et al. 1994; Oslin and Cary 2003; Ritchie and Villebrun 2008). Prevalence can also depend on the age of the population evaluated (i.e., higher prevalence of ARD is found in younger-onset [i.e., ages 45–64] dementia) (Draper et al. 2011*b*; Harvey et al. 2003). MBD appears to be very rare, with only about 250 cases reported between 1966 and 2001 (Helenius et al. 2001).

Human studies offer a full depiction of the consequences of chronic alcohol exposure but are limited by ethical considerations. That is, rigorous experimentation requires the ability to control for relevant variables such as the premorbid condition of the brain. The wide variation (or heterogeneity) of alcoholic populations examined with respect to genetic predisposition, age of onset, pattern of drinking, frequency of withdrawals, length of sobriety, nutritional, and hepatic status has hampered researcher attempts to isolate specific brain regions and mechanisms affected by alcohol, per se. This heterogeneity, and the complexity that it introduces, makes it difficult to thoroughly characterize the disorder. Animal models, in contrast to the indefinite natural course of alcohol use in humans, allow researchers to determine alcohol toxicity in a way that allows them to control for multiple genetic, environmental, and alcohol consumption factors. Animal models permit the study of underlying mechanisms, enabling researchers to better interpret findings from human studies.

On the other hand, animal models also have limitations. Species differences in brain structure and function—among myriad other differences between humans and other animals—can give inadequate information when animal data are applied to human disease. For example, mice models fail to mimic human inflammatory disease with respect to genomic responses (Seok et al. 2013), and corticosteroids disturb development in animals but not in humans (Needs and Brooks 1985). Furthermore, researchers have hypothesized that the design, conduct, and analysis of a mainstay of animal experiments are questionable (Matthews 2008) and rarely undergo meta-analytical review for consensus (Mignini and Khan 2006; Peters et al. 2006; Pound et al. 2004; Sandercock and Roberts 2002).

This article reports key findings in humans, from macrostructural findings using magnetic resonance imaging (MRI), microstructural findings using diffusion tensor imaging (DTI), and metabolic findings from MR spectroscopy (MRS). Studies of alcohol-related central nervous system disorders are used as a framework for findings in uncomplicated alcoholism. The article also examines studies of abstinence and relapse and current imaging studies of animal models of alcoholism and co-occurring brain disorders. The evidence suggests that human studies are necessary to identify and classify the brain systems modified by concomitants of alcoholism versus alcoholism, per se, and that animal models of alcoholism and its co-occurring brain disorders are essential for a mechanistic understanding of vulnerable brain systems.

## Structural MRI

Since the early 1980s, conventional structural MRI has allowed researchers to visualize the living human brain. Detailed images of the brain are possible in part because the different brain tissue types (i.e., gray matter, white matter, and cerebrospinal fluid [CSF]) contain different proportions of water (Rumboldt et al. 2010). With MRI, the brain can be viewed from bottom to top (axial), from front to back (coronal), from left to right (sagittal), or at any oblique angle to these planes. This flexibility also enables greater accuracy in aligning images with internal landmarks, an essential consideration for ensuring consistency of data from replicate images from the same individual (Rohlfing 2006).

### Structural MRI Findings in Alcoholism-Related Brain Diseases

#### Wernicke-Korsakoff Syndrome

**Figure 2:**
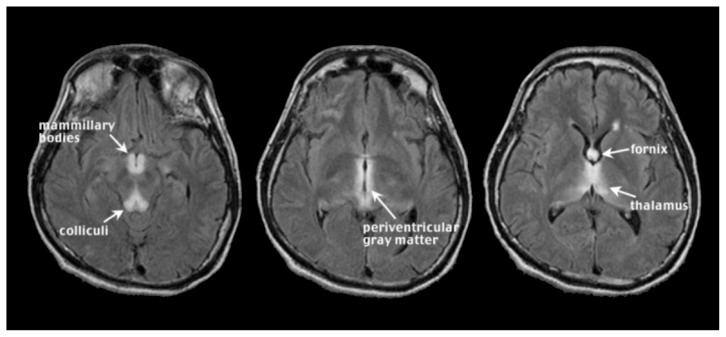
Wernick’s encephalopathy (WE). In acute WE, magnetic resonance imaging (MRI) can detect symmetrical, bilateral hyperintense foci, visible on T2-weighted and fluid attenuation inversion recovery (FLAIR) images, in periaqueductal gray matter, mammillary bodies, and tissue surrounding the third ventricle.

WE occurs with chronic alcoholism and thiamine deficiency. If untreated, WE patients can develop KS, a severe neurological disorder characterized by anterograde amnesia (Harper 2006; Zahr et al. 2011). Malnutrition, vomiting, and diarrhea are common in chronic alcoholism and can contribute to thiamine deficiency (Fields et al. 1994; Gloria et al. 1997; Morgan 1982; Ross et al. 2012). Further, the gastrointestinal tract’s ability to absorb necessary quantities of thiamine is diminished in alcoholics (Hoyumpa 1980; Thomson 2000), and the liver, which houses a large part of the body’s supplies of thiamine, may not be able to store thiamine in the same capacity if it is in a diseased state (Butterworth 2009; Levy et al. 2002). Classical clinical signs of WE included visual, gait, and mental disturbances (Victor et al. 1971), but more recent assessments describe mild, moderate, and severe signs and symptoms including anorexia, loss of memory, and emotional changes (Thomson et al. 2008). An MRI image of acute WE (see figure 2) has symmetrical bright spots, or hyperintensities, clearly visible on T2-weighted images, and those created by fluid attenuation inversion recovery^2^ (FLAIR). The bright spots appear in the midbrain gray matter surrounding the cerebral aqueduct (i.e., periaqueductal gray matter), mammillary bodies, and tissue surrounding the third ventricle^3^ (Lenz et al. 2002; Sullivan and Pfefferbaum 2009). These findings agree with postmortem diagnosis of WE, often requiring evidence of lesions in the mammillary bodies and periventricular areas (e.g., Caine et al. 1997). In addition, observed MR hyperintense areas in WE include the thalamus, cerebellar vermis (Murata et al. 2001), dorsal medulla, tectal plates (Ha et al. 2012), olivary bodies, and dorsal pons (Liou et al. 2012). MRI analysis of KS patients compared with unaffected research participants (i.e., nonalcoholic control subjects) revealed substantial volume shrinkage of the mammillary bodies in KS and a lesser but significant volume deficit in uncomplicated alcoholics (Sheedy et al. 1999; Sullivan et al. 1999*b*; but see Shear et al. 1996; Victor et al. 1989). In contrast with early MR studies suggesting that KS affects the mammillary bodies while sparing the hippocampi (Squire et al. 1990), more recent work demonstrates hippocampal volume deficits in KS (Sullivan and Marsh 2003). Other regions affected by KS are the thalamus, orbitofrontal cortex (Jernigan et al. 1991*b*), cerebellum, and pons (Zahr et al. 2009).

#### Hepatic Encephalopathy (HE)

**Figure 3:**
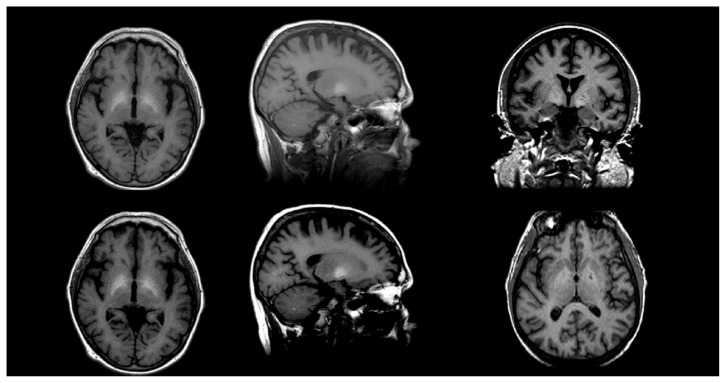
Hepatic encephalopathy (HE). T1-weighted imaging in HE reveals bilateral, symmetrical, high-intensity signals in basal ganglia structures, particularly the globus pallidus and substantia nigra, probably due to manganese deposition and T1 shortening. T2-weighted fluid attenuation inversion recovery (FLAIR) shows hyperintense signals along the corticospinal tract and diffuse hyperintense white matter signal in the cerebral hemispheres.

HE, occurring in acute or chronic liver disease, including acute liver failure and cirrhosis, is believed to arise, at least partially, from high levels of ammonia circulating in the blood. HE patients may appear confused and disoriented and have poor coordination (Prakash and Mullen 2010; Vaquero et al. 2003). T1-weighted images of HE show bilateral, symmetrical, and high-intensity signals in basal ganglia structures, particularly the globus pallidus and substantia nigra (Binesh et al. 2006; Cordoba et al. 2002; Naegele et al. 2000; Pujol et al. 1996; Taylor-Robinson et al. 1995) (see figure 3). T2-weighted FLAIR images show hyperintense signals along the corticospinal tract and diffuse increases in white-matter signal intensities in the cerebral hemispheres (Rovira et al. 2002, 2008). These in vivo MR features correspond with evidence of increased numbers of nonneuronal (i.e., glial) cells called astrocytes in basal ganglia and cerebral cortex of HE brains (Caine et al. 1997). Although discriminating features of WE and HE have been outlined, these diseases can be difficult to differentially diagnose and distinguish, because patients can appear to have similar symptoms and comparable MRI results, especially among alcoholics (Thorarinsson et al. 2011).

#### Central Pontine Myelinolysis (CPM)

**Figure 4:**
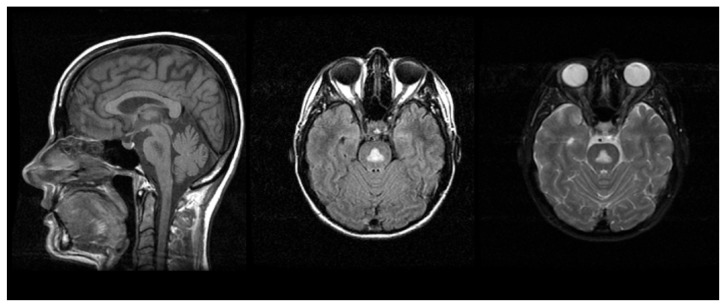
Central pontine myelinolysis (CPM) is visualized as a hypointense T1 (left, sagittal slice) or hyperintense T2 (middle, right axial slices are early and late echo images) symmetric triangle or “bat-wing” lesion in the pons.

A significant proportion of CPM cases (Goebel and Herman-Ben Zur 1976; Messert et al. 1979) include a history of alcoholism. People with CPM, which is associated with electrolyte disturbances and specifically with aggressive correction of low sodium levels in the blood (i.e., hyponatraemia) (Chua et al. 2002), may have symptoms such as the inability to control facial movements, decreased voluntary muscle control (i.e., ataxia), and acute changes in consciousness (Kumar et al. 2006; Pfister et al. 1985). Classically, CPM was characterized by the presence of a symmetric triangular or “bat-wing” lesion in the pons (DeWitt et al. 1984; Gerard et al. 1987), with hypointense T1-weighted (Kleinschmidt-Demasters et al. 2006; Martin and Young 1995) and hyperintense T2-weighted (Buis and Wijdicks 2002; Kleinschmidt-Demasters et al. 2006; Pfister et al. 1985; Martin and Young 1995) images (see figure 4) reflecting damage to the protective covering of nerve cells (i.e., demyelination) as noted postmortem (Goldman and Horoupian 1981). The term osmotic myelinolysis (e.g., Chua et al. 2002; de Souza and Desai 2012) was coined to reflect the fact that other brain regions (e.g., basal ganglia, thalami, and cerebral gray–white matter junctions) are affected in CPM (e.g., Chen et al. 1996; Graff-Radford et al. 2011; Hagiwara et al. 2008; Harlan et al. 1988; Price et al. 1987; Waragai and Satoh 1998), despite suggestions that pathology in these other regions may not strictly represent demyelination (Kleinschmidt-Demasters et al. 2006; Kumar et al. 2006). Because a postmortem study of 112 autopsy cases of CPM patients reported that 28 percent could also be diagnosed with WE (Goebel and Herman-Ben Zur 1976), pontine dysfunction should be regarded as a cardinal clinical sign of CPM.

#### Alcoholic Cerebellar Degeneration (ACD)

ACD patients most frequently display ataxia, although other symptoms can include uncontrollable and repetitive eye movement (i.e., nystagmus) and speech problems resulting from impaired muscle control (i.e., dysarthria) (Fitzpatrick et al. 2012). Neuroimaging in ACD demonstrates damage disproportionately apparent in anterior superior portions of the cerebellar vermis (Sullivan et al. 2000*a*), with postmortem pathology indicating loss of cerebellar Purkinje cells (Feuerlein 1977).

#### Alcohol-Related Dementia (ARD)

Alcoholic dementia, or ARD, a currently preferred term, remains a controversial diagnosis because of confounding syndromes such as WE and HE. Nevertheless, certain clinically distinguishing features of ARD exist. It often occurs in socially isolated men at younger ages of onset (i.e., younger than age 65) than other types of dementia (Draper et al. 2011*a*; Ridley et al. 2013); deficits in visuospatial, executive, and memory functions (Schmidt et al. 2005); slower progression compared with other types of dementia (Gupta and Warner 2008); and partial reversibility (Oslin and Cary 2003). ARD is considered a frontal dementia (Stewart 2006). In support of such categorization, forensic evaluation of a sample of alcoholic brains noted a consistent pattern of synaptic loss in the superior laminae of the frontal cortex (i.e., Brodmann area 10), not related to liver disease (Brun and Andersson 2001).

#### Marchiafava-Bignami Disease (MBD)

MBD, a disease marked by mildly impaired mental status (e.g., confusion) and sometimes by dysarthria (Lee et al. 2011) or ataxia (Arbelaez et al. 2003), is poorly understood but may be related to nutritional deficiencies in addition to chronic alcohol consumption (Kawamura et al. 1985). Traditionally characterized by demyelination and necrosis of the corpus callosum, a number of reports identify cortical lesions in so-called MBD (Ihn et al. 2007; Johkura et al. 2005; Khaw and Heinrich 2006; Namekawa et al. 2013; Tuntiyatorn and Laothamatas 2008; Yoshizaki et al. 2010). Such data, however, represent single case studies and may reflect inaccurate MBD diagnoses. As observed in the pons in CPM, lesions (Clavier et al. 1986) appear hyperintense on T2-weighted images (Bano et al. 2009; Carrilho et al. 2013; Gambini et al. 2003) and hypointense on T1-weighted images (Bano et al. 2009; Carrilho et al. 2013; Kawamura et al. 1985) and often are located along the entire extent of the corpus callosum (Hillbom et al. 2014).

Given the aforementioned findings in clinically differential and diagnosable alcohol-related syndromes, the following section examines whether similar brain disorders also appear in alcoholics who do not manifest the full spectrum of symptoms present in these conditions. That is, how do the brains of uncomplicated alcoholics compare? Quantitative MRI has shown that relatively mild yet significant structural deficits characteristic of alcoholic syndromes can occur in uncomplicated alcoholics.

### Structural MRI Findings in Uncomplicated Alcoholism

**Figure 5:**
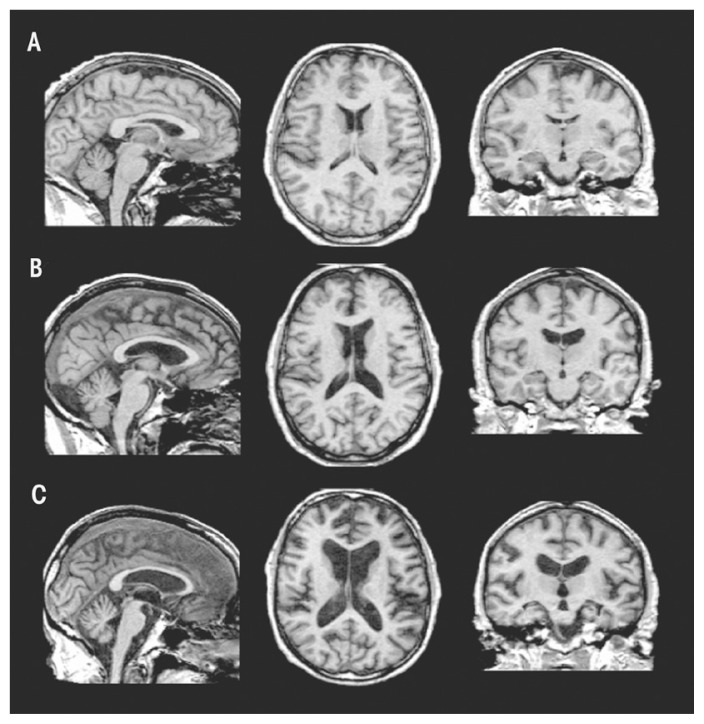
Brain volume deficits in a healthy control **(A)** compared with a subject with uncomplicated alcoholism **(B)** and a subject with Korsakoff’s syndrome (KS) **(C)**. These structural MRIs show a graded effect of volume deficits, notable in the ventricular and sulcal cerebrospinal fluid (CSF)–filled spaces: subjects with KS > subjects with uncomplicated alcoholism > normal controls.

Relative to findings in WKS, research demonstrates mild volume deficits in the mammillary bodies (Shear et al. 1996; Sullivan et al. 1999), hippocampi, and thalami in uncomplicated alcoholics compared with healthy controls (De Bellis et al. 2005; Chanraud et al. 2007; Pitel et al. 2012; Sullivan 2003; van Holst et al. 2012). As shown in figure 5, these structures show a graded effect of volume deficits. That is, volume deficits are greatest in brains of subjects with KS (figure 5C) compared with brains of subjects with uncomplicated alcoholism (figure 5B) and brains unaffected by alcohol (figure 5A). Results suggest that mammillary-body damage is not prerequisite for the development of amnesia in alcoholism (Shear et al. 1996). MR findings also show hippocampal volume deficits in alcoholics compared with healthy controls (Agartz et al. 1999; Beresford et al. 2006; Kurth et al. 2004; Laakso et al. 2000; Sullivan et al. 1995; Wilhelm et al. 2008). Hippocampal volume deficits in alcoholism are influenced by age (Sullivan et al. 1995), even though age-related decline is difficult to detect in cross-sectional studies (Pfefferbaum et al. 2013; Raz et al. 2010; Sullivan et al. 2005*b*). Although deficits in hippocampal volume are not related to seizure incidence (Bleich et al. 2003; Sullivan et al. 1996), temporal-lobe white matter may be sensitive to alcohol-withdrawal seizures (Sullivan et al. 1996). Hippocampal volume shrinkage in alcoholism is attributed to loss of white matter and decreased axonal diameter (Harding et al. 1997). Glial cell loss (Korbo 1999) or reduced incorporation of newly formed neurons to the dentate gyrus (He et al. 2005; Nixon and Crews 2004), however, could also affect hippocampal volume in alcoholism.

Other regions selectively affected in WE and KS include the orbitofrontal cortices (KS), periaqueductal gray matter, and tissue surrounding the third ventricle (WE). Reports suggest that propensity to relapse following sobriety is related to pronounced atrophy in bilateral orbitofrontal cortices (Beck et al. 2012; Cardenas et al. 2011; Durazzo et al. 2011; also see Rando et al. 2011). The third ventricle (i.e., enlargement) is sensitive to resumption of chronic alcohol consumption (Pfefferbaum et al. 2001; Sullivan et al. 2000*b*). There currently are no studies regarding periaqueductal gray-matter volume in uncomplicated alcoholics.

Key regions affected in HE include the globus pallidus and substantia nigra. Volume effects on these two structures have not been reported in uncomplicated alcoholics; however, in children with fetal alcohol syndrome, globus pallidus volume is reduced in size compared with unaffected children (Nardelli et al. 2011). In contrast, other basal ganglia nodes of reward circuitry have been described as affected in uncomplicated alcoholism (Durazzo et al. 2011; Makris et al. 2008): MRI studies have revealed smaller volumes of caudate (Boutte et al. 2012), putamen (Jernigan et al. 1991*a*), amygdala (Fein et al. 2006), and nucleus accumbens, especially in more recently sober alcoholics compared with healthy controls (Sullivan et al. 2005*a*). Given the role of the amygdala in emotional regulation and behavioral control (for review, see McBride 2002), however, researchers have speculated that premorbid amygdala volume deficits put individuals at heightened risk for developing AUD (Benegal et al. 2007; Clarke et al. 2008; Kamarajan et al. 2006).

**Figure 6:**
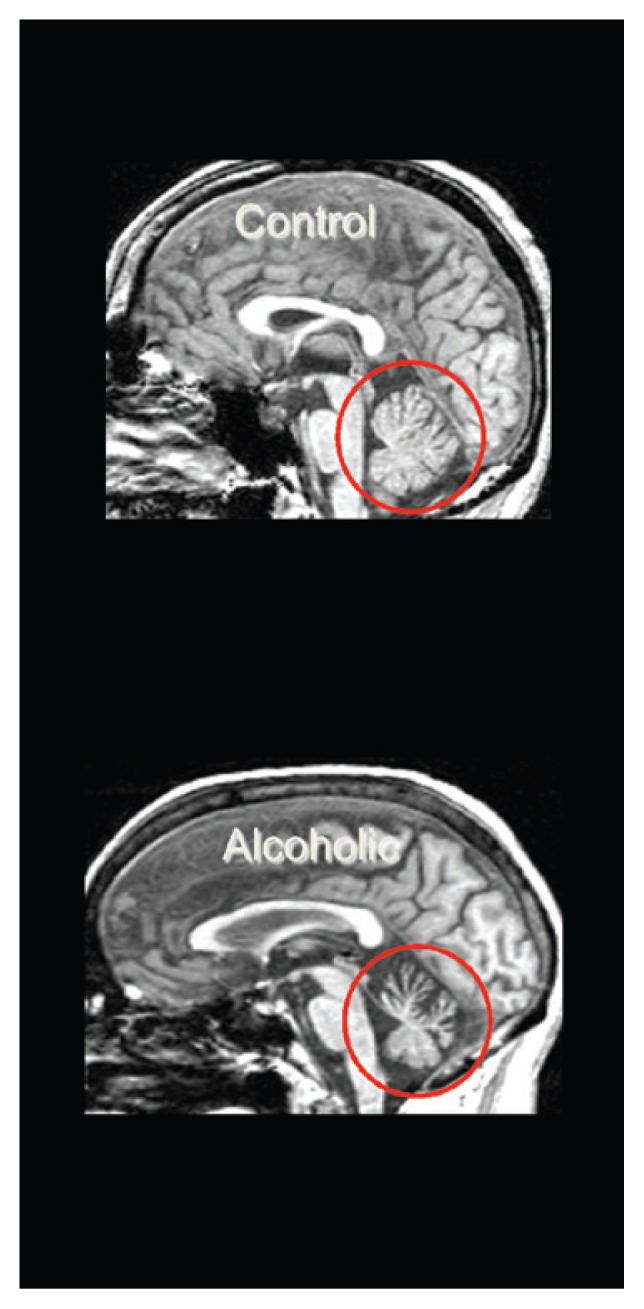
Cerebellar volume deficits in uncomplicated alcoholism. Midsagittal view of the brain, showing smaller volume of the anterior superior vermis of the cerebellum in an alcoholic man (bottom) compared with an age-matched control man (top).

CPM targets the pons and ACD affects the cerebellum. Total infratentorial volume (including pons, cerebellar hemispheres, vermis, fissures, cisterns, and fourth ventricle) is significantly smaller in uncomplicated alcoholics than control subjects. The volume of the pons (Chanraud et al. 2009*b*; Pfefferbaum et al. 2002*b*; Sullivan 2003) and cerebellum (i.e., hemispheres) (Boutte et al. 2012; Chanraud et al. 2007, 2009*a*; De Bellis et al. 2005; Sullivan et al. 2000*a*,*c*) is smaller in uncomplicated alcoholics than in normal controls. Alcoholism-related volume deficits are also prevalent in gray and white matter (Shear et al. 1996; Sullivan et al. 2003) of the cerebellar vermis (Antunez et al. 1998; Piguet et al. 2006; Sullivan et al. 2006*b*, 2010), predominately in anterior superior but not posterior inferior regions (Sullivan et al. 2000*a*) (see figure 6).

The frontal cortex is notably damaged in ARD. With respect to cortical regions in uncomplicated alcoholism, various methods have shown significant, widespread shrinkage of both cortical gray and white matter with corresponding increases in CSF-filled spaces (Cardenas et al. 2007; Jang et al. 2007; Jernigan et al. 1991*a*; Mechtcheriakov et al. 2007; Pfefferbaum 1992). In particular, older (older than age 50) but not younger adult alcoholics show disproportionate deficits in both gray- and white-matter cortical volume, especially in the frontal lobes, when volumes are statistically adjusted for brain tissue decline associated with normal aging (Cardenas et al. 2005, 2007; Pfefferbaum et al. 1997). This is the case even in comparisons made in groups selected on alcohol consumption, where older alcoholics have consumed equivalent amounts over their lifetime as younger alcoholics.

Thinning of the corpus callosum occurs in uncomplicated alcoholics and is more prominent in the anterior than posterior regions (Estruch et al. 1997; Pfefferbaum et al. 1996). As with WE and KS, evidence for MBD-like pathology in uncomplicated alcoholism raises the possibility that brain damage occurs on a continuum. The following section examines how brain structures and function respond when drinking stops.

### Structural MRI Findings in Recovery From Alcoholism

Longitudinal MRI investigations show that the ventricles become smaller following weeks (Schroth et al. 1988; Zipursky et al. 1989) or months (Shear et al. 1994) of drinking cessation. Reduction of lateral ventricles precedes reduction of third-ventricular volume (Pfefferbaum et al. 1995) and may be related to improvements in hematocrit, hemoglobin, and red blood cell counts (Pfefferbaum et al. 2004). The following brain structures increase in volume in response to abstinence: the entire cerebral cortex (Liu et al. 2000); temporal, insular, and anterior cingulate cortices (Cardenas et al. 2007); amygdala (Wrase et al. 2008) (a finding that would argue against a premorbid volume deficit); thalamus (Cardenas et al. 2007); hippocampus (Liu et al. 2000, Wrase et al. 2008); brainstem; and cerebellar cortex (Cardenas et al. 2007; Liu et al. 2000).

Sober alcoholics reveal several associations between brain-volume gain, as determined by MRI, and improvement in neuropsychological test performance: Reduced lateral-ventricle volume is related to improved memory performance (Rosenbloom et al. 2007), reduced third-ventricle volume is related to improved nonverbal short-term memory performance (Sullivan et al. 2000*b*), and reduced fourth-ventricle volume is related to improvement in measures of ataxia (Rosenbloom et al. 2007).

The brain’s capacity to return to “normal” following long-term sobriety is unknown. Short-term (6 weeks) abstinence seems sufficient to observe some brain-volume recovery but does not result in equivalent brain volumes between recovering chronic alcoholics and healthy controls (Mann et al. 2005). It is difficult to determine whether recovery is complete. Aging is a factor. That is, older alcoholics exhibit reduced capacity for recovery compared with younger alcoholics (Fein et al. 1990; Munro et al. 2000; Reed et al. 1992; Rourke and Grant 1999). Longer periods of abstinence may be required for follow-up investigations. Some brain damage, such as neuronal loss (Harper 2007), may be irreversible, even with extended abstinence.

Despite evidence for recovery of brain volume with abstinence, the mechanisms accounting for recovery remain unclear. One hypothesis, brain rehydration, was not supported by early human research studies (Schroth et al. 1988). An alternative explanation suggests that new neurons are created (i.e., neurogenesis) (e.g., Mandyam and Koob 2012): It is unlikely, however, that enough neurons could be made to replace the volume loss observed in chronic alcoholism. Nor is it clear that new neurons can migrate from neurogenic zones to distant areas of volume loss (Rakic 2002). On the other hand, adequate volume recovery may be explained by white-matter regeneration, because glial cells (i.e., oligodendroctyes) have the capacity to repair myelin and remyelinate neurons (Kipp et al. 2012), and oligodendrocyte progenitor cells have the potential to migrate long distances (Tirotta et al. 2010). Indeed, alcoholics who relapse have decreased white matter (Pfefferbaum et al. 1995), whereas continued abstinence is associated with increased white matter (Shear et al. 1994), notably in the corpus callosum and subcortical white matter (Cardenas et al. 2007).

Turning from studies with humans to animals, the following section examines imaging studies in models of alcoholism and related disorders.

## Using Animal Models and Structural MRI to Study Alcoholism-Related Brain Disease

### WE

There are two experimental approaches to model WE in rodents. The slower approach uses a thiamine-deficient diet (i.e., feeding with a thiamine-deficient chow), which can take 3–4 weeks to produce symptoms. Behavioral symptoms can be achieved in ~2 weeks using a combination of a thiamine-deficient chow and intraperitoneal (i.p.) administration of a thiamine pyrophosphokinase inhibitor such as pyrithiamine (Hazell and Butterworth 2009). Both models result in symptoms that mimic those observed in humans with WE (Pitkin and Savage 2001). Structural MRI findings in thiamine-deficient animals show similar patterns of brain changes, including hyperintense signals observed on T2-weighted images in thalamus, collicular bodies (Dror et al. 2010; Jordan et al. 1998; Pfefferbaum et al. 2007; Zahr et al. 2014*a*), hypothalamus, hippocampus (Jordan et al. 1998), mammillary bodies (Pfefferbaum et al. 2007), corpus callosum, and superior cerebellar peduncles (Dror et al. 2010). Thiamine deficiency may cause degeneration through neuroinflammatory mechanisms (Abbott 2000; Hazell and Butterworth 2009). In rats, inflammatory genes were highly expressed in vulnerable brain regions (Vemuganti et al. 2006). MRI in animal models permits further probing of the effects of thiamine deficiency on the brain and can be used to determine susceptible brain regions as a function of time of insult (Dror et al. 2010; Zahr et al. 2014*a*) as well as relationships between neuroinflammatory markers and brain insult (Zahr et al. 2014*a*). Such studies have also been used to confirm a mechanism of toxicity suspected based on research in humans (e.g., Harper 1980; Koguchi et al. 2004; Navarro et al. 2008): that glucose loading in a thiamine-deficient state can precipitate WE (Jordan et al. 1998; Zahr et al. 2014*a*), likely involving a breakdown of the blood–brain barrier (Nixon et al. 2008; Zelaya et al. 1995). In animals, postmortem followup can be used to confirm and extend in vivo findings. For example, electron microscopy showed a higher percentage of small fibers and myelin thinning in the corpus callosa of thiamine-deficient animals relative to controls (He et al. 2007).

Research with animals demonstrates that thiamine deficiency impairs several biochemical pathways requiring the thiamine derivative thiamine pyrophosphate (e.g., transketolase, pyruvate dehydrogenase, and α-ketoacid dehydrogenase) (Thomson et al. 2012), thereby interfering with carbohydrate metabolism (for energy production), lipid metabolism (for production and maintenance of myelin), and amino acid metabolism (for production of glucose-derived neurotransmitters; for example, glutamate and γ-aminobutyric acid [GABA]) (Sechi and Serra 2007; Vetreno et al. 2012). Consequently, the function of essential thiamine-requiring enzymes in the brain (e.g., transketolase, pyruvate dehydrogenase, and α-ketoacid dehydrogenase) is compromised, leading to oxidative stress, cellular energy impairment, and eventually neuronal loss (Thomson et al. 2012).

Evidence also shows that thiamine deficiency alters norepinephrine, dopamine (Mousseau et al. 1996), serotonin (Nakagawasai et al. 2007), and histamine (Langlais et al. 2002; McRee et al. 2000) synthesis and catabolism pathways. Thiamine deficiency may target focal brain areas such as the thalamus because, relative to other brain structures, it has lower levels of monocarboxylic acid transporters and acetyl-CoA-synthetase. This makes these areas less capable of generating energy from acetate (Qin and Crews 2014), which is a potential source of cellular energy in place of glucose in alcoholism (Volkow et al. 2013).

Current rodent models to study HE include models of acute and chronic liver failure (Butterworth et al. 2009; Diaz-Gomez et al. 2011). According to the International Society for Hepatic Encephalopathy, however, “At this time, there are no satisfactory animal models of Type C HE resulting from end-stage alcoholic liver disease or viral hepatitis, the most common etiologies encountered in patients” (Butterworth et al. 2009, p. 783). In addition, no MR-imaging studies to date have used rodent models of HE. Imaging studies in cats, dogs, and monkeys (Moon et al. 2012; Torisu et al. 2005; Zhou et al. 2012) typically recapitulate the human condition, showing nonspecific sulcal widening and hyperintensities in lentiform nuclei (i.e., putamen and globus pallidus of the basal ganglia) (Torisu et al. 2005; Zhou et al. 2012). Animal models of HE have been used to evaluate potential mechanisms of pathology, such as the contribution of excess ammonia in the blood (i.e., hyperammonemia) (Cauli et al. 2014) or lactate (Bosoi et al. 2014). Animal models of HE have also been used to explore treatment strategies for HE (e.g., hypothermia) (Barba et al. 2008).

### CPM

Using a rat model of CPM to study white-matter degeneration, it was found that blood–brain barrier breakdown, detected with MRI, was associated with a higher risk of developing demyelination, as detected using postmortem histopathology (Adler et al. 2000). This study demonstrated that blood–brain barrier disruption exposes oligodendrocytes to substances normally excluded from the brain. This supports hypotheses from human postmortem studies suggesting that damage to the pons may be linked to reduced blood flow, as indicated by findings that basilar artery architecture is altered in CMP (De Reuck et al. 1975).

Although there are no known studies using structural MRI in animal models of ACD, ARD, or MBD, the following section examines animal studies in uncomplicated alcoholism.

## Structural MRI Findings in Animal Models of Uncomplicated Alcoholism

An important initial report in the rodent MRI literature was the demonstration that brain growth continues beyond what would be considered adulthood in rats bred to prefer alcohol (i.e., alcohol-preferring rats, or P rats). Indeed, whole-brain volume in such rats continued to grow until approximately postnatal day 450 (Sullivan et al. 2006*a*), well past adulthood, which is typically considered as postnatal day 90 (Bell et al. 2013). Baseline studies (in the absence of alcohol [i.e., EtOH] exposure) also suggest that brains of alcohol-preferring rats are different relative to their wild-type counterparts, including reduced gray-matter volume in thalamus, ventral tegmental area, and insular and cingulate cortices (Gozzi et al. 2013).

One of the most consistent findings in alcohol-exposed rodents, ventricular enlargement, varies with timing and method of alcohol exposure. It is far more pronounced in rats achieving average blood alcohol levels (BALs) of 250 mg/dL in just 4 days of involuntary binge-type administration of EtOH (Zahr et al. 2010, 2013, 2014*b*) than in rats achieving average BALs of 200 mg/dL over 24 weeks using vapor EtOH exposure (Pfefferbaum et al. 2008) or P rats gradually achieving average BALs of 125 mg/dL with voluntary EtOH consumption (Pfefferbaum et al. 2006*a*), where only modest ventricular enlargement was noted (cf., Fadda and Rossetti 1998; Nixon 2006). Even repeated binge exposures (i.e., 5 cycles of 4 days of intragastric binge EtOH exposure with 1 week abstinence in between), do not result in persistent effects on the brain detectable with MRI (Zahr et al. 2015). Although ventricular size increases with each binge EtOH exposure, there is rapid recovery during each week of abstinence (Zahr et al. 2015). Such studies suggest that EtOH alone, at least in the exposure protocols evaluated with MRI, does not result in the characteristics observed in human alcoholics. Conversely, rats exposed to vaporized EtOH during adolescence are reported to show persistent effects (i.e., ventricular enlargement and deficits in hippocampal volume) into adulthood (Ehlers et al. 2013; Gass et al. 2014). Mice exposed to EtOH during adolescence are similarly purported to exhibit long-lasting regional brain-volume deficits in the olfactory bulb and basal forebrain (Coleman et al. 2011, 2014). These results suggest that the adolescent rodent brain may be more vulnerable to enduring toxic effects of EtOH than the adult rodent brain.

In monkeys trained to voluntarily consume alcohol, those that drank at least 3 g/kg EtOH per day for 15 months showed significant brain-volume shrinkage in the cerebral cortices (Kroenke et al. 2014). Because these animals were well nourished, these results suggest a direct relationship between oral EtOH intake and measures of decreased brain gray-matter volume.

## Microstructural DTI

A number of sources provide extensive descriptions of the principles of DTI (Basser and Jones 2002; Chien et al. 1990; Gerig et al. 2005; Jones 2005; LeBihan 2001, 2003; Pierpaoli et al. 1996; Poupon et al. 1999; Sullivan and Pfefferbaum 2011). Briefly, DTI takes advantage of the fact that MR images of the brain are predominantly maps of water protons with contrast created by their immediate environment and their motility. In regions with few or no constraints imposed by physical boundaries, such as CSF in the ventricles, water movement is random and uniform in every direction and is therefore isotropic. In contrast to CSF, the path of a water molecule along a white-matter fiber is constrained by physical boundaries such as the axon sheath, causing greater movement along the long axis of the fiber than across it. This movement is called anisotropic; diffusion along the long axis of a fiber (axial or longitudinal diffusion) is greater than diffusion across the fiber (radial or transverse diffusion) (Song et al. 2002).

DTI findings are described in terms of diffusion. The magnitude of diffusion, referred to as mean diffusivity (MD) or the apparent diffusion coefficient (ADC), is calculated mathematically. Increased MD corresponds to white-matter damage. Fractional anisotropy (FA), ranging between 0 and 1, reflects axonal integrity, with lower integrity reflected by FA values closer to 0. Thus, disruption of white-matter microstructure detectable with DTI can reflect compromised myelin, cytoskeletal structure, or axonal density (Basser 1995; Basser and Pierpaoli 1996; Spielman et al. 1996).

Several approaches have been used to quantify DTI metrics. One of the more desirable approaches is the use of quantitative fiber tracking, which is able to evaluate fibers along their entire length and can thus detect compromised white matter. This technique can be used to depict selective commissures (e.g., corpus callosum), projection fibers, and association fibers.

### DTI Findings in Alcoholism-Related Brain Disorders

In one study, DTI in alcoholics with (*n* = 7) and without (*n* = 20) WKS showed FA deficits in the fornix and cingulum bundle of the Papez or medial limbic circuit, measured using tract-based spatial statistics (TBSS). These FA effects were greater in alcoholics with WKS relative to those without it (Segobin et al. 2015). The number of tracts in the fornix appears to be reduced only in WKS patients (Nahum et al. 2015).

Studies of people who have alcohol-related cirrhosis with HE have reported elevated MD in several white-matter bundles, including the corpus callosum, internal capsule, and frontal white matter (Kale et al. 2006), and effects on both FA and MD of occipital white matter (Kumar et al. 2008). HE caused by alcoholism compared with other forms of HE (e.g., as a result of viral infection or primary biliary cirrhosis) appears to have different effects on DTI parameters (Miese et al. 2006), with more widespread changes in FA and MD in alcoholic relative to nonalcoholic cirrhosis (Ahluwalia et al. 2015). When researchers induced hyperammonemia in cirrhotic patients, an increase in ADC in brain white matter was observed, supporting excess ammonia in the blood as a mechanism driving cerebral edema (Mardini et al. 2011).

DTI showed elevated MD in the middle cerebellar peduncles with no effects on corticospinal tracts in a study participant with CPM relative to three healthy comparison participants (Min et al. 2012; Nair et al. 2012).

The largest DTI study of MBD to date included six study participants, five with a history of chronic alcoholism. All six showed hyperintense signals on diffusion images and low ADC of the corpus callosum. Researchers observed cortical lesions in frontoparietal regions in three of six study participants with the poorest outcomes (Menegon et al. 2005). Remaining DTI studies of MBD were case studies (e.g., Tuntiyatorn and Laothamatas 2008) showing low ADC along the entire corpus callosum (Bano et al. 2009; Wenz et al. 2014), with FA values diminishing progressively from front to back (Pacheco et al. 2014; Sair et al. 2006). No known DTI studies have been conducted in patients with ACD or ARD.

### DTI Findings in Uncomplicated Alcoholism

DTI has revealed microstructural damage related to alcoholism in cerebral areas that appear intact in structural MRI analyses (e.g., Pfefferbaum and Sullivan 2002; Pfefferbaum et al. 2006*b*; Sullivan et al. 2003). Corpus callosum findings in uncomplicated alcoholics are common and, as observed for MBD, show greater anterior than posterior effects (e.g., Arnone et al. 2006; Konrad et al. 2012; Liu et al. 2010; Pitel et al. 2010; Schulte et al. 2005). Quantitative fiber tracking has demonstrated greater FA deficits in anterior than in posterior fibers of supratentorial and infratentorial white-matter bundles in alcoholics compared with healthy controls, as well as low FA in tracts of the corpus callosum, centrum semiovale, internal and external capsules, fornix, superior cingulate, and longitudinal fasciculi (Fortier et al. 2014; Müller-Oehring et al. 2009; Pfefferbaum and Sullivan 2005; Pfefferbaum et al. 2000, 2002*a*, 2009*a*; Trivedi et al. 2013). Frontolimbic (Harris et al. 2008; Monnig et al. 2013), fronto-parietal (Maksimovskiy et al. 2014), fronto-occipital (Bagga et al. 2014), fronto-cerebellar (Sullivan and Pfefferbaum 2005), cortico-striatal (Yeh et al. 2009), and cortico-pontine (Chanraud et al. 2009*b*) fibers are also affected in alcoholics relative to healthy controls.

Studies have also examined DTI- function relationships in alcoholism. FA in anterior cingulate and motor areas correlates with executive and psychomotor performance (Konrad et al. 2012), FA in the splenium correlates with working memory (Pfefferbaum et al. 2000), and FA in several regions (corpus callosum, parietal, occipital, and frontal white-matter) correlates with performance on the Iowa Gambling Task (Zorlu et al. 2013). A double dissociation was found showing that higher diffusivity in sensory- motor and parietal bundles was associated with poorer balance but not psychomotor speed, whereas higher diffusivity in prefrontal and temporal bundles was associated with slower psychomotor speed but not balance (Pfefferbaum et al. 2010). DTI changes in multiple supratentorial and infratentorial fiber systems in alcoholics correlated with impairment in speeded performance and postural stability (Pfefferbaum et al. 2009*b*), frontal fiber integrity connecting left and right hemispheres predicted performance on a coordinated psychomotor task (Rosenbloom et al. 2008), and number of reconstructed fibers running between the pons and the midbrain was related to cognitive flexibility performance (Chanraud et al. 2009*b*). Gray-matter diffusivity in the hippocampus, which is lower in alcoholics than in healthy controls, is related to episodic memory impairment (Chanraud et al. 2009*a*).

### DTI Findings in Recovery from Alcoholism

Similar to structural MRI findings demonstrating pronounced tissue- volume shrinkage of orbitofrontal cortices in abstinent alcoholics who were likely to resume drinking (e.g., Beck et al. 2012; Cardenas et al. 2011; Durazzo et al. 2011), DTI identified alcoholic individuals more likely to resume drinking 6 months following initial evaluation based on lower FA and higher diffusivity in frontal white matter at baseline (Sorg et al. 2012). Increases in FA and decreases in diffusivity have been interpreted as evidence for white-matter recovery with abstinence. Studies have shown recovery in corpus callosum at 1 year compared with 2 weeks of abstinence (Alhassoon et al. 2012) and in frontal white matter at 1 month compared with 1 week of abstinence, at least in nonsmoking, sober alcoholics (Gazdzinski et al. 2010). Other reports suggest that some white-matter impairments persist after 6 to 30 months of recovery in alcoholics relative to healthy controls (Zorlu et al. 2014). In a seminal longitudinal study of 47 alcoholic and 56 healthy controls study participants, Pfefferbaum and colleagues (2014) reported that, despite abnormally low FA, age trajectories of the alcoholics who abstained were positive and progressing toward normality, whereas those of the relapsing alcoholics and control subjects were negative.

### DTI Findings in Animal Models of WE

DTI data have been collected in animal models of WE but not in other concomitants of alcoholism.

In the study in which WE was induced by thiamine deficiency, animals were imaged at baseline, presymptomatic stage (day 10), symptomatic stage (days 12 and 14), and after recovery on days 31 and 87. A decrease in FA in the inferior colliculi was first noted on day 10 but showed recovery on day 87. On the other hand, the FA decrease in the thalamus first noted on day 12 persisted through day 87 (Dror et al. 2010). This model was also used in a pharmacological DTI study in which animals were exposed to rasagiline, a selective monamine oxidase B inhibitor, as a potential protective agent against thiamine-deficiency–induced brain damage (Dror et al. 2014). In addition to reducing ventricular enlargement, rasagiline appeared to ameliorate the effects of thiamine deficiency on the FA decrease in the thalamus (Dror et al. 2014). Histopathology showed that treatment with rasagiline reduced the lesions in thalamus and colliculi observed in the thiamine-deficient brain (Eliash et al. 2009). Rasagiline has not been evaluated in human patients with WE.

### DTI Findings in Animal Models of Uncomplicated Alcoholism

Adolescent animals exposed to intermittent EtOH and evaluated postmortem showed no effects on FA but reduced axial diffusivity (hippocampus, cortex, and cerebellum), reduced radial diffusivity (hippocampus and cortex), and reduced MD (cerebellum and corpus callosum) in several brain regions (Vetreno et al. 2016). Adult rats exposed to a single dose of EtOH showed a slight and transient reduction, relative to unaffected rats, in ADC in brainstem (Kong et al. 2013), frontal lobe, hippocampus, thalamus, and cerebellum (Liu et al. 2014). These findings were interpreted as reflecting the development of cytotoxic brain edema, as histological analysis showed cell swelling and narrowed extracellular spacing (Kong et al. 2013).

Whereas chronic exposure to vaporized EtOH did not result in detectable effects on FA or MD, binge EtOH exposure resulted in transient decreases in FA and transient increases in MD (Pfefferbaum et al. 2015). Together, these results suggest that DTI can detect acute and subchronic effects on the brain, but that chronic exposure to EtOH can result in brain adaptations such that effects on FA and MD are no longer discernable.

## Magnetic Resonance Spectroscopy

Although MRI primarily depicts the distribution of water protons, similar technology can also be used to obtain information about chemical constituents other than water, primarily due to a small frequency shift, or “chemical shift,” relative to the water signal. The acquisition of MR-detectable signals other than those of water and fat is referred to as MRS and is an in vivo application of traditional laboratory-based NMR spectroscopy.

MRS reveals information about several biochemicals, or metabolites, in the brain. The largest signals arise from *N*-acetylaspartate (NAA), creatine and phosphocreatine (i.e., total creatine [tCr]), and choline-containing compounds (Cho). Signals from the combined resonances of glutamate (Glu) and glutamine (Gln) (i.e., Glx) are also sometimes reported, as are myo-inositiol (mI) and lactate (lac). Signals from Glu and GABA can also be detected under certain conditions.

### MRI and Signals for Four Prominent Metabolites

#### NAA

The predominant in vivo proton signal is NAA, with contributions from other *N*-acetyl compounds, especially *N*-acetyl aspartyl glutamate. NAA is found almost exclusively in neurons (Petroff et al. 1995; Urenjak et al. 1992, 1993) and, thus, is considered a measure of neuronal integrity. Postmortem (Cooper 1972; Koller et al. 1984; Nadler and Cooper 1972) and MRS (Kwo-On-Yuen et al. 1994; Petroff et al. 1995) studies have shown NAA levels to be higher in gray than in white matter in healthy study participants, as have in vivo studies (Doyle et al. 1995; Lim and Spielman 1997; Lim et al. 1998; Moyher et al. 1995; Narayana et al. 1989; Pouwels and Frahm 1998; Schuff et al. 1999; Wang et al. 1998).

#### tCr

The tCr signal, generated by creatine and phosphocreatine, is influenced by the state of high-energy phosphate metabolism (Tedeschi et al. 1995). In spectroscopy studies, it often is used as a reference for other peaks based on the incorrect assumption that its concentration is relatively constant (cf. Zahr et al. 2008, 2009, 2014*b*).

#### Cho

The in vivo MRS-visible Cho peak is generated primarily by water-soluble choline-containing compounds (free choline, phosphocholine, and glycerophosphocholine) (Barker et al. 1994) and is associated with cell-membrane synthesis and turnover. The Cho resonance also provides an index of cellular density in brain tumors (Gupta et al. 1999) and may be a marker of increases in glial density with age and disease. MRS-measured Cho concentration is higher in white than gray matter (Pfefferbaum et al. 1999) and increases with normal aging (Chang et al. 1996; Kreis et al. 1993; Moats et al. 1994; Pfefferbaum et al. 1999; Soher et al. 1996).

#### mI

Myo-inositiol is present in glial but not neuronal cell cultures (Brand et al. 1993; Petroff et al. 1995) and plays a role in maintaining cell volume (Ernst et al. 1997; Lien et al. 1990). The concentration of mI is higher in gray than in white matter (Michaelis et al. 1993; Pouwels and Frahm 1998).

**Figure 7:**
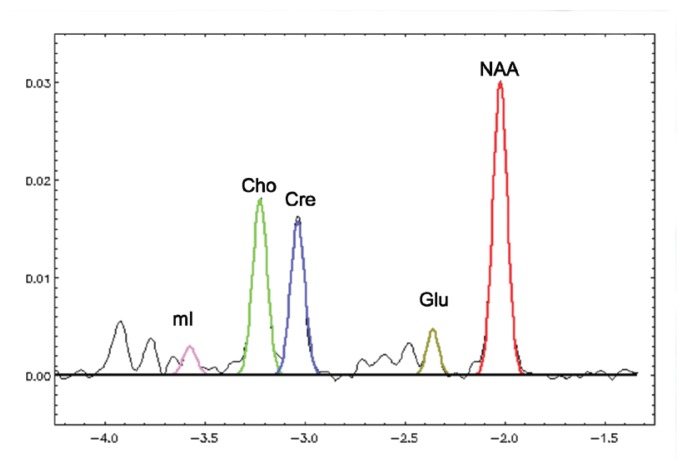
Magnetic resonance spectroscopy spectra from the thalamus of a 55-year-old nonalcoholic control woman, with a gaussian fit of the major metabolites that has been color coded.

Figure 7 shows a graph of MR spectra from the thalamus of a 55-year-old nonalcoholic woman. The major metabolites are color coded.

### MRS Findings in Alcoholism-Related Brain Disorders

As with the other imaging modalities, MRS reports of WE are primarily case studies. For example, a Japanese man who had consumed alcohol for 50 years and had eaten poorly for several days as a result of a cold presented with gait disturbances and incoherent speech. MRS before and after thiamine treatment found an initial low level of NAA/tCr in the thalamus, which appeared to increase with thiamine replacement. NAA/tCr levels in the cerebellum did not increase, although a lactate peak initially present in the cerebellum resolved (Murata et al. 2001). MRS conducted in two patients with non–alcohol-related thiamine deficiency (i.e., caused by gastric and pancreatic cancer) compared with five healthy study participants showed similar results: relatively low NAA/tCr levels in the thalamus resolved after treatment with thiamine (Mascalchi et al. 2002).

In a variety of brain regions, MRS findings in alcohol-related cirrhosis and HE are remarkably consistent and comparable with findings in nonalcoholic HE (e.g., Cordoba et al. 2001; Gupta et al. 1993; Häussinger et al. 1994), showing lower levels of Cho/tCr and mI/tCr and higher levels of Gln/tCr (Ahluwalia et al. 2015; Binesh et al. 2006; Chavarria et al. 2013; Jain et al. 2013; Kreis et al. 1992; Laubenberger et al. 1997; Miese et al. 2006; Pujol et al. 1996; Singhal et al. 2010; Taylor-Robinson et al. 1994, 1999; Thomas et al. 1998). Levels of mI and Cho are lowest and Glx highest in patients with HE (Geissler et al. 1997; Lee et al. 1999; Poveda et al. 2010; Ross et al. 1994; Tarasow et al. 2003).

Mild swelling of astrocytes is proposed as the key event in the pathogenesis of HE (e.g., Takahashi et al. 1991). In cirrhosis, elevated blood level of ammonia is thought to result in elevated brain ammonia, which can be toxic (Weissenborn et al. 2007). It often has been proposed that the brain’s response to elevated ammonia levels is to combine ammonia and glutamate to make glutamine using glutamine synthetase, found primarily in astrocytes (Yamamoto et al. 1987). Thus, brain swelling in cirrhosis is thought to reflect an increase in astrocytic glutamine formation. The decrease in mI is thought to be a compensatory mechanism to counterbalance the osmotic effect of cerebral glutamine accumulation (Balata et al. 2003; Mardini et al. 2011). Although some articles claim to measure in vivo glutamine (e.g., Binesh et al. 2006; Chavarria et al. 2013; Jain et al. 2013; Kreis et al. 1992; McConnell et al. 1995), it is unlikely that the MRS method used in these cases permitted the separate detection of glutamate and glutamine, which are strongly coupled and difficult to detect independently, even with very short echo times (Adalsteinsson et al. 2002). A single study measured GABA levels in five alcoholics without HE and five study participants with both alcohol and non–alcohol-related HE. GABA levels were lower in the two patient groups relative to 10 comparison participants (Behar et al. 1999).

In the only report of MRS conducted in a case of alcoholism-associated CPM, elevated Cho/tCr was found and interpreted as reflecting edema or demyelination in a 53-year-old man with gait disturbances and hearing loss (Nomoto et al. 2004). In 12 patients with chronic hyponatremia (nonalcohol etiology), MRS showed reduced Cho and mI relative to unaffected study participants, reflecting osmolyte disturbances (Videen et al. 1995).

Research also has found compromised NAA/tCr levels in patients with cerebellar degeneration (Tedeschi et al. 1996; Terakawa et al. 1999). Two MRS case studies of MBD showed reduced NAA/tCr and elevated Cho/tCr in corpus callosum splenium (Gambini et al. 2003; Tuntiyatorn and Laothamatas 2008), findings consistent with demyelination (elevated Cho) and axonal injury (reduced NAA).

### MRS Findings in Uncomplicated Alcoholism

Most MRS studies show lower levels of NAA in recently sober alcoholics relative to healthy controls in several brain regions, including frontal areas (Bendszus et al. 2001; Durazzo et al. 2004, 2010; Fein et al. 1994; Jagannathan et al. 1996; Meyerhoff et al. 2004; Schweinsburg et al. 2003; Seitz et al. 1999) and cerebellum (Bendszus et al. 2001; Durazzo et al. 2010; Jagannathan et al. 1996; Parks et al. 2002; Seitz et al. 1999). Similarly, studies in AUD patients shortly following detoxification have found low levels of Cho (Bendszus et al. 2001; Durazzo et al. 2004; Ende et al. 2005; Fein et al. 1994; Parks et al. 2002; Seitz et al. 1999), although Cho findings in AUD are less consistent (e.g., Hermann et al. 2012; Modi et al. 2011). Because these findings are prominent in white matter, it is thought that the effects of alcoholism are greater in white than in gray matter (De la Monte 1988; Harper et al. 2003).

### MRS Findings in Recovery from Alcoholism

MRS studies suggest that NAA (e.g., Bartsch et al. 2007; Bendszus et al. 2001; Parks et al. 2002), particularly in frontal (Bartsch et al. 2007; Bendszus et al. 2001; Durazzo et al. 2006) and cerebellar (Bendszus et al. 2001; Fein et al. 1994; Parks et al. 2002) regions and Cho levels (e.g., Bartsch et al. 2007; Bendszus et al. 2001; Durazzo et al. 2006; Ende et al. 2005; Martin et al. 1995) show normalization (i.e., increase) with abstinence. Elevations in mI are not seen in long-term sober alcoholics (Schweinsburg et al. 2000). These findings suggest that low NAA levels initially observed in recently sober alcoholics reflect neurodegeneration without cell death, and increases with abstinence may reflect healing without cell generation. The disruption and recovery of Cho and mI levels suggest white-matter recovery with sobriety and the potential for remyelination.

### MRS Findings in Animal Models of Syndromes Associated With Alcoholism

In rat models of WE induced using pyrithiamine, the dominant MRS pattern is a reduction in both NAA and Cho in several brain regions (Lee et al. 1995, 2001; Rose et al. 1993), including the thalamus (Navarro et al. 2008, 2005; Pfefferbaum et al. 2007). Researchers also frequently report elevations in lactate (Navarro et al. 2005, 2008). Precipitation of WE with glucose (resulting in seizures) is associated with further decreases in NAA and Cho and, significantly, an elevation in lactate (Zahr et al. 2014*a*). Treatment with thiamine is associated with recovery in Cho levels (Lee et al. 1995).

MRS has been used to evaluate models of HE achieved using various methods (Cudalbu 2013) and most reports show similar findings. Hepatic devascularization (Barba et al. 2008; Zwingmann et al. 2004), carbon tetrachloride treatment (Bates et al. 1989), bile-duct ligations (Bosoi et al. 2014; Rackayova et al. 2015), and other means of promoting hyperammonemia (e.g., acute liver ischemia, urease, or methionine sulfoximine treatment) (Bosman et al. 1990; de Graaf et al. 1991) result in elevated levels of Gln and frequently lactate in rat brain (e.g., cortex). Additional effects reported include lower levels of NAA, mI, Cho, and Glu (Barba et al. 2008; Bates et al. 1989; Bosman et al. 1990; de Graaf et al. 1991; Peeling et al. 1993; Rackayova et al. 2015; Zwingmann et al. 2004). As in the human condition, a similar caveat holds: that is, it is not clear if Glu and Gln are clearly discriminated in many of these studies, and, often, reports more likely reflect Glx levels.

Although in vivo MRS studies in both humans and animals have persisted in interpreting elevations in brain Gln as reflecting elevations in peripheral ammonia and brain edema (Venkatasubramanian et al. 2001), ex vivo carbon 13 nuclear MR studies have challenged the convention that glutamine accumulation is the major cause of brain edema in acute HE. Such studies instead indicate limited metabolic pathway reactions and capacity of astrocytes to detoxify ammonia by glutamine synthesis and emphasize distortions of energy and neurotransmitter metabolism (Zwingmann 2007).

### MRS Findings in Animal Models of Uncomplicated Alcoholism (and Recovery)

MRS can be used in animals to detect and quantify in vivo and real-time brain EtOH kinetics (e.g., rats: Sullivan et al. 2005*c*; monkeys: Kaufman et al. 1994). Unlike findings in long-term sober human alcoholics, nonabstinent chronic heavy drinkers (Meyerhoff et al. 2004) and social and moderate drinkers (Ende et al. 2006) show elevated levels of brain Cho. Elevated levels of Cho are also reported in the thalamus of rodents between weeks 16 and 40 of alcohol exposure (Lee et al. 2003). Neuroimaging research has been conducted with rodent models of binge (Zahr et al. 2010, 2013, 2014*b*), repeated binge (Zahr et al. 2015), and chronic alcohol exposure (Zahr et al. 2009). In vivo MRS studies have consistently shown that a single 4-day binge exposure with BALs approaching 300 mg/dL is associated with reversible changes to the brain: levels of NAA are lower and those of Cho are higher following binge EtOH exposure (Zahr et al. 2010, 2013, 2014*b*). In the repeated-binge experiment, animals were exposed to 5 cycles of 4 days of intragastric EtOH treatment and 10 days of recovery. Changes in MRS metabolite levels again were transient: levels of NAA decreased, whereas those of Cho increased with each binge EtOH exposure cycle but then recovered during each abstinence period. Changes in response to EtOH were in expected directions based on the previous, single-binge EtOH exposure experiments but did not accrue with repeated-binge EtOH exposure (Zahr et al. 2015). In the chronic EtOH exposure study, NAA levels were lower in the EtOH-exposed relative to the comparison group but did not attain statistical significance, whereas levels of Cho appeared to demonstrate a dose-response curve (i.e., increasing levels with higher and longer EtOH exposure) (Zahr et al. 2009).

## Conclusion

Imaging investigations of alcohol-related brain disorders show unique neuropathology (as outlined in table 1), offering a framework for examining pathology in uncomplicated alcoholism. Because brains affected by AUD can show mild effects in the regions aggressively targeted by overt disease, animal models have been useful in distinguishing the etiology of pathology and differentiating brain regions specifically targeted by thiamine deficiency versus hyperammonemia, for example. Individuals with AUD may show more prominent effects in some regions compared with others, suggesting a propensity for one diagnosis over another (e.g., an alcoholic may be more vulnerable to thiamine deficiency than to liver damage). What remains unresolved, and what animal models can help determine, is why certain brain regions are differentially vulnerable to certain pathologies. For example, are the colliculi sensitive to thiamine deficiency because of their relatively high metabolic rate (Landau et al. 1955; Sokoloff et al. 1977)? Is the pons susceptible to CPM because of its proximity to the basilar artery? Does dopamine explain why basal ganglia are targets of liver disease (Mousseau et al. 1993)?

In vivo imaging studies in humans and animal models will continue to provide an evolving picture of the course of alcoholic brain disease through remissions and exacerbations as long-term studies follow human alcoholics as they age and as new initiatives evaluate adolescents before they are exposed to alcohol.

## Figures and Tables

**Figure 1:**
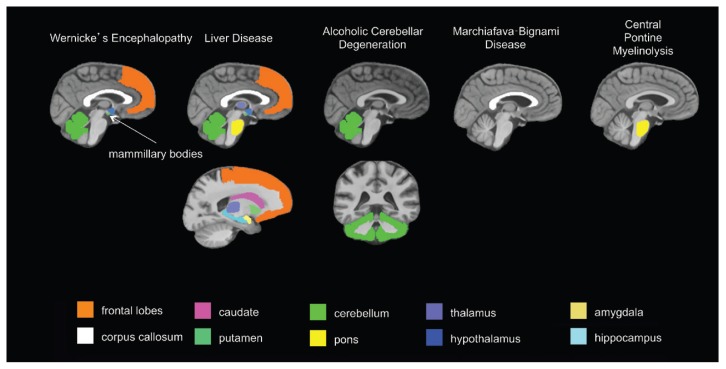
Brain regions targeted by alcoholism-related diseases.

**Table 1:** Radiological Signatures in Brain Imaging of Patients with Alcoholism-Related Syndromes

Alcoholism-Related Syndrome	Abbreviation	Primary Targeted Region(s)	Secondary Targeted Regions	Prevalence in Alcoholics (Percentage)
**Wernicke’s Encephalopathy**	WE	Mammillary bodies, periaqueductal gray matter, dorsal medulla, tectal plates, olivary bodies, pons, tissue surrounding 3rd ventricle		12–18
**Korsakoff’s Syndrome**	KS	Mammillary bodies, hippocampus, thalamus, orbitofrontal cortices	Cerebellum, pons	10–15
**Hepatic Encephalopathy**	HE	Globus pallidus, substantia nigra	Corticospinal tract, cortex	3–16
**Central Pontine Myelinolysis**	CPM	Pons	Basal ganglia, thalamus, cerebral gray–white matter junctions	< 0.5
**Alcoholic Cerebellar Degeneration**	ACD	Cerebellum		0.4–42
**Alcohol-Related Dementia**	ARD	Frontal cortex		3–24
**Marchiafava-Bignami Disease**	MBD	Corpus callosum	Cortex	< 0.002

## Footnotes

1 WE and KS are neurological disorders caused by thiamine deficiency. HE is a decline in brain function as a result of severe liver disease. CPM is a neurological disorder resulting from the destruction of the myelin layer that covers nerve cells in the pons. ACD occurs when neurons in the cerebellum are damaged due to alcohol use. MBD is a neurological disease associated with alcoholism, caused by damage to the corpus callosum.

2 Researchers use different MRI techniques to highlight different aspects of the brain. Techniques mentioned in this article include T1 weighted, T2 weighted, and FLAIR.

3 The cerebral aqueduct and third ventricle are part of the brain’s ventricular system—a set of cavities in the brain that produce, transport, and remove cerebrospinal fluid. This system also includes the lateral ventricles and fourth ventricle.
